# 

**Published:** 1918-08-17

**Authors:** 


RATES OF SUBSCRIPTION:

Twelve months, 10/10; Six months, 5/5; Three months, 2/9, post free to any address in the United Kingdom. Abroad, Twelve months, 13/-; Six months, 6/6; Three months, 3/3, post free. **THE HOSPITAL "Index" to Volume LXI., October 19 7 to March 1918, is now ready, price 3½d. per copy, post free.** Address The Manager, THE HOSPITAL, "The Hospital" Building, 28/29 Southampton Street, Strand, London, W.C. 2.

"LYSOL" EVANS

*(Also known as "EVANSOL")*

*Neutral and Non-Corrosive.*

*In 4-oz., 8-oz., 16-oz. and 32-oz. Bottles.*

*Specify "LYSOL" EVANS and ensure a standard article that can be completely relied upon.*

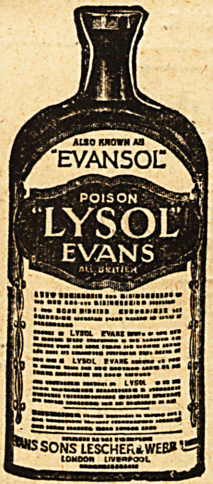

*Gives a Clear Solution.*

*In ½-gall and 1 gall Tins.*

*On application we will send Free a full 4-oz. Sample to Medical Men, Nurses and Institutions.*

*Evans Sons Lescher & Webb, Limited*

*60 Bartholomew Close, London.* *56 Hanover Street, Liverpool.*

A

### HOSPITAL SUNDAY FUND'S RECORD COLLECTIONS.

### £85,000 Distributed.

We are indebted to the *Times* for Sir Ernest Tritton's speech at the meeting of the Council of the Metropolitan Hospital Sunday Fund, held at the Mansion House on the 9th inst. :—

Sir Ernest Tritton, who presided, said that the council was to be congratulated upon a "record" year for subscriptions. The previous best year had been exceeded by £15,000. This was largely due to a gift of £5,000 from the American Red Cross Society. On behalf of the council he expressed its high appreciation of such a gift from such a quarter—a proof of the sympathy existing between the United States and Great Britain in their common effort on behalf of humanity and civilisation. An increased number of clergy and ministers had reponded to the council's request not to rely this year on collections in churches and chapels, but to carry the claims of hospitals to the homes of rich and poor throughout the parishes by letter or by house-to-house collection. From St. Mark's, North Audley Street, the amount received was £2,453; never before had such a sum been received from one London church; St. Andrew's, Lambeth, a very poor parish, by collection in pence raised £76. So rich and poor had shown what could be done in response to the suggestion of the council that collection should not be restricted to those attending public worship. People were willing to give to the hospitals if they had the opportunity; the poor had given heartily, for they knew what benefit they received from the hospitals. If these efforts were followed up energetically the much-desired total of £100,000 would be reached before long. The council rejoiced to hand over such a sum as £85,000 for the benefit of hospitals where so many of our gallant wounded men were tended.

It was proposed by the Venerable Archdeacon of London, Canon Holmes, seconded by Mr. J. R. Cooper, and resolved (Prebendary Eardley Wilmot alone dissenting): That the Report of the General Purposes Committee, upon the question of Hospitals and Sunday Kinematograph Shows, be, and is hereby received and adopted, and that the best thanks of the council are accorded to the committee for the time and trouble they have given to the matter.

### ANNUAL REPORT OF THE COMMITTEE OF DISTRIBUTION.

The Committee of Distribution held their first meeting on Monday, June 3, and then, and at subsequent meetings, examined the reports and income and expenditure accounts of the several institutions seeking to participate, and made their awards in conformity with the Laws of the Constitution.

The Committee beg to report that the total of the Fund on August 15 will amount to £85,652.

This year 238 institutions have applied to participate in the Fund, being three less than in 1917, and the Committee now recommend the distribution of £85,038 6s. 7d. to the several hospitals, institutions, dispensaries, and nursing associations shown in the appendix hereto.

The Committee having reduced, or postponed the determination of the bases of award to sixteen institutions, the governing bodies of the same were informed thereof in accordance with the Laws of the Constitution. Eight deputations attended the Committee, and after hearing their several explanations, and those of the remainder having been received by letter, the bases of award were finally determined.

Seven and a-half per cent. of the total sum available for distribution is appropriated to the purchase of surgical appliances during the ensuing year, and 2½ per cent. for district nursing associations.

In compliance with an order of the council, statistics have been prepared as usual for the use of its members, showing the number of beds in hospitals, the cost of patients at hospitals and dispensaries, the proportionate expense of management to maintenance, and other information. A copy thereof has been sent to each member of the council.

C. A. Hanson, Lord Mayor, President and Treasurer.
C. Ernest Tritton, Vice-Chairman.
William S. Church, Chairman of Committee.

| | |
|---|---|
| W. Hardy Harwood. | G. H. Makins. |
| Somerleyton. | Adrian Pollock. |
| John C. Bell. | Marcus Samuel. |
| F. A. Bevan. | W. Hale White. |

C. C. Wakefield, John Henry Buxton, } Hon. Secs.

The Mansion House, August 1, 1918.

### The Report of the Committee of Distribution for the Year 1918 was approved, with the following Awards.

#### THIRTY-FIVE GENERAL HOSPITALS.

Charing Cross Hospital, £1,989; French Hospital, £47 5s.; Great Northern Central Hospital; £1,948 10s.; Guy's Hospital, £3,467 5s.; Hampstead and N.W. London Hospital, £1,066 10s.; Italian Hospital, £133 17s. 6d.; Kensington General Hospital, £186 15s.; King's College Hospital, £2,466; King Edward's, Ealing, £189; London Hospital, £10,217 5s.; London Homœopathic Hospital £829 2s. 6d.; London Temperance Hospital, £751 10s.; Metropolitan Hospital, £1,468 2s. 6d.; Middlesex Hospital and Convalescent Home, £3,730 10s.; Mildmay Hospital, £327 7s. 6d.; Mildmay Memorial Hospital, £111 7s. 6d.; Miller Hospital and Royal Kent Dispensary, £792; Phillips' Memorial Homœopathic Hospital, £60 15s.; Poplar Hospital, £541 2s. 6d.; Prince of Wales' Hospital, £1,332; Royal Free Hospital, £2,047 10s.; St. Andrew's, Dollis Hill, £36; St. George's Hospital, £3,235 10s.; SS. John and Elizabeth Hospital, £119 5s.; St. John's Hospital, Lewisham, £267 15s.; St. Mary's Hospital, £2,199 7s. 6d.; St. Thomas's Hospital, £1,138 10s.; Seamen's Hospital Society, £1,072 2s. 6d.; University College Hospital, £1,994 12s. 6d.; Walthamstow, etc., Hospital, £198; Wandsworth, Bolingbroke Hospital, £525 7s. 6d.; West Ham Hospital, £1,342 2s. 6d.; West London Hospital, £1,798 17s. 6d.; Westminster Hospital, £1,643 12s. 6d.; Wimbledon Hospital, £63.

#### SIX CHEST HOSPITALS.

City of London Hospital for Diseases of the Chest, Victoria Park, £1,333 2s. 6d.; Hospital for Consumption, Brompton, £2,899 2s. 6d.; Mount Vernon Consumption Hospital, £157 10s.; Royal Hospital for Diseases of the Chest, £425 5s.; Royal National Sanatorium, £25 17s. 6d.; Royal National Hospital, Ventnor, £281 5s.

#### TWENTY CHILDREN'S HOSPITALS.

Alexandra Hospital for Hip Disease, £121 10s.; Banstead Surgical Home, £45; Barnet Home Hospital, £34 17s. 6d.; Belgrave Hospital, £416 5s.; Cheyne Hospital for Incurable Children, £241 17s. 6d.; East London Hospital for Children, £1,019 5s.; Evelina Hospital for Sick Children, £339 15s.; Home for Incurable Children, £49 10s.; Home for Sick Children, Sydenham, £214 17s. 6d.; Hospital for Sick Children, £1,644 15s.; Infants' Hospital, Westminster, £356 12s. 6d.; Kensington, for Children, and Dispensary, £87 15s.; Paddington Green Hospital for Children, £454 10s.; Queen's Hospital for Children, £1,902 7s. 6d.; Royal Waterloo Hospital for Children and Women,

£592 17s. 6d.; St. Mary's Hospital, Plaistow, £363 7s. 6d.; St. Monica's Hospital, Brondesbury, £110 5s.; Victoria Hospital for Children, Chelsea, £802 2s. 6d.; Victoria Home, Margate, £33 15s.; Hospital for Hip Disease, Sevenoaks, £56 5s.

NINE LYING-IN HOSPITALS.

British Home for Mothers and Babies, £38 5s.; City of London Lying-in Hospital, £313 17s. 6d.; Clapham Maternity Hospital and Dispensary, £45; East End Mothers' Home, £154 2s. 6d.; General Lying-in Hospital, £317 5s.; Jewish Maternity, £100 2s. 6d.; Plaistow Maternity Hospital, £87 15s.; Queen Charlotte's Lying-in Hospital, £722 5s.; Salvation Army Lying-in Hospital, £60 15s.

SIX HOSPITALS FOR WOMEN.

Chelsea Hospital for Women, £483 15s.; Hospital for Women, £493 17s. 6d.; Grosvenor Hospital for Women, £195 15s.; New Hospital for Women, £616 10s.; Samaritan Free Hospital, £676 2s. 6d.; South London Hospital, £262 2s. 6d.

TWENTY-TWO OTHER SPECIAL HOSPITALS.

Middlesex Hospital, Cancer Wing, £334 2s. 6d.; London Fever Hospital, Islington, £281 5s.; St. Mark's Hospital for Fistula, £495; National Hospital for the Diseases of the Heart, £218 5s.; Female Lock Hospital, £532 2s. 6d.; Male Lock Hospital, £39 7s. 6d.; Hospital for Epilepsy, Paralysis, etc., £425 5s.; National Hospital for the Paralysed and Epileptic, £1,328 12s. 6d.; West End Hospital for Diseases of the Nervous System, £424 2s. 6d.; Central London Ophthalmic Hospital, £302 12s. 6d.; Royal Eye Hospital, £285 15s.; Royal London Ophthalmic Hospital, £1,276 17s. 6d.; Royal Westminster Ophthalmic Hospital, £256 10s.; Western Ophthalmic Hospital, £149 12s. 6d.; Royal National Orthopædic Hospital, £526 10s.; Royal Sea Bathing Hospital, Margate, £112 10s.; St. John's Hospital for Diseases of the Skin, £56 5s.; St. Peter's Hospital for Stone, £202 10s.; Central London Throat and Ear Hospital, £60 15s.; Throat Hospital, Golden Square, £164 5s.; Metropolitan Ear, Nose, and Throat Hospital, £77 12s. 6d.; Royal Dental Hospital of London, £190 2s. 6d.

TWENTY-SIX CONVALESCENT HOSPITALS.

Metropolitan Convalescent Institution, Walton, £450; Metropolitan Convalescent Institution, Broadstairs, £67 10s.; Metropolitan Convalescent Institution, Bexhill, £706 10s.; All Saints' Convalescent Hospital, Eastbourne, £73 2s. 6d.; All Saints' Convalescent Home, St. Leonards, £31 10s.; Ascot Priory Convalescent Home, £50 12s. 6d.; Brentwood Convalescent Home for Children, £21 7s. 6d.; Chelsea Hospital for Women, Convalescent Home, St. Leonards, £50 12s. 6d.; Mrs. Gladstone's Convalescent Home, Mitcham, £56 5s.; Friendly Societies' Convalescent Home, Dover, £84 7s. 6d.; Hahnemann Convalescent Home, Bournemouth, £22 10s.; Hanwell Convalescent Home, £7 17s. 6d.; Hastings, Fairlight Sanatorium, £50 12s. 6d.; Herne Bay Baldwin-Brown Convalescent Home, £56 5s.; Homœopathic Hospital Convalescent Home, £5 12s. 6d.; Hemel Hempstead Convalescent Home, £64 2s. 6d.; Mary Yolland Home, £23 12s. 6d.; Police Seaside Home, Brighton, £90; St. Andrew's Convalescent Home, Clewer, £45; St. Andrew's Convalescent Home, Folkestone, £157 10s.; St. John's Home, Kemp Town, £28 2s. 6d.; St. Joseph's Convalescent Home, Bournemouth, £14 12s. 6d.; St. Leonards-on-Sea Convalescent Home, £157 10s.; Eversfield, St. Leonard's, £18; St. Mary's Convalescent Home, Birchington, £49 10s.; St. Mary's Convalescent Home, Broadstairs, £177 15s.; St. Michael's Convalescent Home, Westgate, £16 17s. 6d.; Seaside Convalescent Hospital, Seaford, £33 15s.

TWENTY-FOUR COTTAGE HOSPITALS.

Acton Cottage Hospital, £132 15s.; Beckenham Cottage Hospital, £121 10s.; Blackheath and Charlton Cottage Hospital, £133 17s. 6d.; Brentwood Cottage Hospital, £43 17s. 6d.; Bromley, Kent, Cottage Hospital, £119 5s.; Canning Town Cottage Hospital, £181 2s. 6d.; Chislehurst, Sidcup, and Cray Cottage Hospital, £117; Coldash Cottage Hospital, £56 5s.; East Ham Cottage Hospital, £45; Eltham Cottage Hospital, £74 5s.; Enfield Cottage Hospital, £111 7s. 6d.; Epsom and Ewell Cottage Hospital, £67 10s.; Hendon Cottage Hospital, £70 17s. 6d.; Hounslow Cottage Hospital, £30 7s. 6d.; Livingstone, Dartford Cottage Hospital, £162; Kingston Cottage Hospital, £66 7s. 6d.; Reigate and Redhill Hospital, £247 10s.; Sidcup Cottage Hospital, £48 7s. 6d.; Surbiton Cottage Hospital, £45; Teddington Cottage Hospital, £22 10s.; Tilbury (Passmore Edwards) Cottage Hospital, £56 5s.; Willesden Cottage Hospital, £122 12s. 6d.; Wimbledon (Nelson) Hospital, £75 7s. 6d.; Wood Green Cottage Hospital, £118 2s. 6d.

ELEVEN INSTITUTIONS.

Florence Nightingale Hospital for Invalid Gentlewomen, £183 7s. 6d.; St. Saviour's Hospital and Nursing Home, £76 10s.; Stoke Newington Home Hospital, £56 5s.; Firs Home, Bournemouth, £16 17s. 6d.; St. Columba's Hospital, £337 10s.; St. Luke's House, Pembridge Square, £158 12s. 6d.; Santa Claus Home, Highgate, £94 10s.; Royal Mineral Water Hospital, Bath, £28 2s. 6d.; Winifred House, Holloway, £67 10s.; Highgate Convalescent Home, £52 17s. 6d.; Holt Children's Sanatorium, £15 15s.

FORTY DISPENSARIES.

Battersea Provident Dispensary, £159 15s.; Billingsgate Dispensary, £58 10s.; Brixton, etc., Dispensary, £55 2s. 6d.; Buxton Street Dispensary, £11 5s.; Camberwell Provident Dispensary, £48 7s. 6d.; Child's Hill Provident Dispensary, £4 10s.; City Dispensary, £74 5s.; Clapham General and Provident Dispensary, £25 17s. 6d.; Eastern Dispensary, £33 15s.; East Dulwich Provident Dispensary, £55 2s. 6d.; Farringdon General Dispensary, £40 10s.; Finsbury Dispensary, £79 17s. 6d.; Forest Hill Provident Dispensary, £36; Greenwich Provident Dispensary, £27; Hampstead Provident Dispensary, £41 12s. 6d.; Islington Dispensary, £42 15s.; Islington Medical Mission, £45; Kilburn, Maida Vale, and St. John's Wood Dispensary, £37 2s. 6d.; Kilburn Provident Medical Institution, £48 7s. 6d.; London Dispensary, Spitalfields, £14 12s. 6d.; London Medical Mission, Endell Street, £81; Margaret Street, for Consumption, £21 7s. 6d.; Metropolitan Dispensary, £27; Notting Hill Provident Dispensary, £23 12s. 6d.; Paddington Provident Dispensary, £19 2s. 6d.; Public Dispensary, £48 7s. 6d.; Queen Adelaide's Dispensary, £12 7s. 6d.; Royal General Dispensary, £24 15s.; Royal Pimlico Provident Dispensary, £24 15s.; St. George's, Hanover Square, Dispensary, £24 15s.; St. John's Wood Provident Dispensary, £36; St. Marylebone General Dispensary, £67 10s.; St. Pancras and Northern Dispensary, £55 2s. 6d.; Stamford Hill, Stoke Newington Dispensary, £49 10s.; Wandsworth Common Provident Dispensary, £9; Westbourne Provident Dispensary, £13 10s.; Western Dispensary, £43 17s. 6d.; Western General Dispensary, £78 15s.; Westminster General Dispensary, £30 7s. 6d.; Woolwich Provident Dispensary, £34 17s. 6d.

THIRTY-THREE NURSING ASSOCIATIONS.

Belvedere, Abbey Wood, £9; Brixton, £36; Central St. Pancras, £45; Charlton and Blackheath, £9; Chelsea and Pimlico, £18; Hackney, £63; Hammersmith, £81; Hampstead, £27; Isleworth, £18; Kensington, £81; Kilburn, £9; Kingston, £45; Lambeth Road (Catholic), £18; Metropolitan (Bloomsbury), £63; St. Olave's (Bermondsey), £36; Paddington and Marylebone, £63; Plaistow, £72; Plaistow (Maternity), £72; Ponders End, Enfield, etc., £18; Rotherhithe, £27; Shoreditch, £72; Sick Room Helps Society, £9; Sidcup, £9; Silvertown, £18; South London (Battersea), £54; Southwark, £36; South Wimbledon, £27; Tottenham, £27; Westminster, £36; Woolwich, £63; East London, £135; North London, £81; Ranyard Nurses, £513.

The best thanks of the council were tendered to the Committee of Distribution for the care they had bestowed upon the work entrusted to them, and also to the Editors of the newspapers who pleaded the needs of the hospitals, and advocated the cause of the Fund.

The meeting closed with the cordial thanks of the council to the Rt. Hon. Sir Charles Augustin Hanson, Bt., M.P., Lord Mayor, the President and Treasurer of the Fund. The vote of thanks to Sir Ernest Tritton for his chairmanship and services to the Fund was carried by acclamation.

